# Development and validation of a simplified BRASS index to screen hospital patients needing personalized discharge planning

**DOI:** 10.1007/s11606-018-4405-y

**Published:** 2018-04-16

**Authors:** Adriana Zarovska, Andrea Evangelista, Tiziana Boccia, Giovannino Ciccone, Daniela Coggiola, Antonio Scarmozzino, Daniela Corsi

**Affiliations:** 1 Department of Medical Sciences, University of Turin, Turin, Italy; 2 Unit of Clinical Epidemiology and Center for Oncologic Prevention (CPO) Piemonte, AOU Città della Salute e della Scienza, Turin, Italy; 3 Continuity of Care Department, AOU Città della Salute e della Scienza, Turin, Italy; 4 Hospital Management of the health care professions, AOU Città della Salute e della Scienza, Turin, Italy; 5 Department of Health Management, AOU Città della Salute e della Scienza, Turin, Italy

**Keywords:** BRASS index, Blaylock Risk Assessment Screening Score, patient discharge, continuity of patient care, discharge planning, hospital mortality

## Abstract

**Background:**

Discharge planning is an important component of hospital care. The Blaylock Risk Assessment Screening Score (BRASS) index is an instrument used to identify patients requiring complex discharge planning.

**Objectives:**

(1) Evaluate the ability of the original BRASS index to predict the risk of complex discharge and hospital mortality. (2) Develop and validate a simplified BRASS index by eliminating redundant variables and re-estimating the predictor weights.

**Design:**

Prospective cohort study.

**Participants:**

Patients admitted at the general internal medicine wards of tertiary referral hospital in Turin, Italy, and screened within 48 h using the BRASS index.

**Methods:**

The first phase of the study assessed the performance of the original BRASS index in predicting the risk of complex discharge and hospital mortality, then a simplified score was developed. In the second phase, temporal validation of the simplified BRASS index was performed. The probability of each discharge modality (discharged at home without complications, complex discharge, and dead in hospital) was modeled using polytomous logistic regression. The AUC was used to compare the performance of the different models.

**Key Results:**

Among 6044 patients in the first phase of the study, 63% were discharged at home without complications, 31% had complex discharge, and 6% died during the hospital stay. The AUC of the simplified BRASS index, compared with the original index were 0.71 vs. 0.70 for complex discharge and 0.83 vs. 0.80 for hospital mortality. In the validation set (3325 patients), the simplified BRASS index discriminates the outcome categories with an AUC of 0.69 and 0.81 for complex discharge and hospital mortality, respectively.

**Conclusion:**

The new, simplified BRASS index showed a slightly better performance in predicting the risk of complex discharge and hospital mortality than the original tool and takes less time to be applied. These results were also confirmed in the validation set.

**Electronic supplementary material:**

The online version of this article (10.1007/s11606-018-4405-y) contains supplementary material, which is available to authorized users.

## INTRODUCTION

Discharge planning is an important component of hospital care. It has been defined as a series of events that occur shortly after a person is admitted to a health care setting with the aim of facilitating continuity of care, reducing length of hospital stay, reducing unplanned readmission to hospital, ensuring the optimal use of hospital beds, and improving the coordination of services following hospital discharge.^1^^,^^2^

A systematic review suggests that a discharge plan tailored to each individual patient probably brings a small reduction in hospital length of stay and of the risk of readmission to hospital for older people admitted for different medical conditions. 1 Therefore, it is important to identify factors that make a patient’s discharge problematic or complex so that the discharge planning and specific actions can be undertaken in timely manner.

According to Selker et al., in the general internal medicine wards, 30% of all hospital discharges can be delayed for non-medical reasons. Among the most frequent causes of delays are the lack or inadequacy of discharge planning and unavailability of post-discharge facilities.^3^ Another study reported that 13.5% of all hospital days were judged unnecessary for acute general medicine inpatient care. Of these unnecessary hospital days, the majority were attributable to either weakness in discharge planning or to difficulties in the placement of the patients in a skilled facility.^4^

Due to different patient social profiles, functional status, and health needs, standardized procedures for early identification of patients at risk of a difficult discharge are widely performed. However, there is no generally accepted screening tool with satisfactory validity and ease of use for this purpose.

Several studies have developed risk screening instruments containing a list of patient characteristics known to be predictive of encountering discharge problems. 5^–^8 These risk screening instruments vary a lot in the outcomes considered (need of complex discharge planning, hospital length of stay (LOS), readmission to hospital, etc.), but none specifically considered the risk of hospital mortality as a correlated or competing outcome.

The BRASS index (Blaylock Risk Assessment Screening Score) is a screening tool used at hospital admission to identify patients at risk of prolonged hospitalization requiring specific discharge planning. The BRASS index was used and validated for the first time on a group of older hospitalized patients in the Netherlands, by Blaylock and Cason in 1992.^8^

The BRASS index consists of several items organized in 10 domains: age, living situation/emotional support, functional status, cognition, behavior pattern, mobility, sensory deficits, previous admissions/ER (emergency room) visits, number of active medical problems, and drugs, with values ranging between 0 (lowest risk) and 30 (highest risk). This index was introduced in the year 2012 in the Piedmont region (Italy) with an official deliberation for obligatory use in all public hospitals. In the San Giovanni Battista hospital in Turin, a cutoff point of 13 or higher was used for activating the special discharge planning service.

Although the BRASS index has been used in different settings, indications and groups of patients,^9^^–^11 the practical experience suggested that this screening instrument could be improved by simplifying and re-weighting its items. The specific objectives of this study were (1) to evaluate the ability of the original BRASS index in predicting both the risk of a complex discharge (CD) and the correlated risk of hospital mortality (HM); (2) to develop a simplified BRASS index by eliminating redundant variables and re-estimating the predictor weights; and (3) to validate the new, simplified BRASS index.

## METHODS

### Study Design

This is a prospective cohort study including all patients admitted at the general internal medicine wards of the San Giovanni Battista Hospital, Città della Salute e della Scienza in Turin, Italy. Our study consisted of two phases. In the first (development phase), we assessed the ability of the original BRASS index in predicting the risk of complex discharge and the correlated event of hospital mortality, then a new simplified score was developed by eliminating the redundant variables and re-weighting those retained. In the second (validation phase), we validated the new simplified BRASS index in a temporal validation set.

The training set population comprised patients discharged by the general internal medicine wards of the San Giovanni Battista Hospital between 1st January 2015 and 31st December 2015. The validation cohort included patients discharged by the same wards between the 1st of January 2016 and 30th of June 2016. In both study phases, all patients were screened within 48 h of admission with the BRASS index by trained nurses. The interview collected demographic, social (age, living situation/social support), and clinical information (cognitive status, mobility, sensory deficit, behavior pattern and number of previous admissions, active medical problems, and drugs). Patients were asked to report their functional status by evaluating a few activities of daily living (ADLs) and instrumental activities of daily living (IADLs) which were rated dichotomously as dependent or independent in each activity. The data regarding the discharge modality, the destination, and the length of stay (LOS) were retrieved from the hospital discharge records (SDO) of the patients.

### Outcomes

There is no widely accepted definition of complex discharge. We classified the discharge modality (primary study outcome) into three categories:

- Discharged at home without complications, including all patients discharged at home with a length of stay (LOS) lower than the 90th percentile of DRG-specific LOS observed in Piedmont region hospitals.
- Complex discharge, including all alive patients not discharged at home or patients discharged at home with a LOS greater than the 90th percentile of DRG-specific LOS observed in Piedmont region hospitals in the same year.
- Dead in hospital.

### Statistical Analysis

The probabilities of the three discharge modalities were modeled using a polytomous (multinomial) logistic regression analysis using the training set population. Firstly, we evaluated the predictive ability of the BRASS index as a single continuous variable. The linearity of the BRASS index on the probability of each discharge modality was evaluated using a restricted cubic spline transformation of the continuous variable. The discrimination ability of the model to distinguish individuals with different discharge modalities was evaluated using the area under the ROC curve (AUC). The revision of the original BRASS index was performed according to the different importance of each item (predictor). Before performing the regression model, a data reduction (predictor selection) was made by removing items with very low prevalence (< 20 cases) or those strongly associated with other items (using a graphical representation of the hierarchical clustering of the variables and applying both practical and clinical judgments to select those to be removed). To confirm the predictivity of the variables removed by those retained, the AUCs were estimated using logistic (for dichotomous items) and ordinal logistic (for ordinal items) regression models. The final model was fitted using a backward selection, by eliminating one by one those candidate predictors based on Akaike information criterion (AIC) and choosing the final model with the lowest AIC.

The final, simplified model was used to derive two separate scores to better predict the two different outcomes: (a) complex discharge and (b) hospital mortality. The scores were based on regression coefficients, converted into integer numbers proportionally to the lowest coefficient satisfying two conditions: an OR > = 1.20 and a *p* value < 0.10. The updated and simplified BRASS index was calculated for each patient using the new scoring rule.

In the second phase, the simplified BRASS index was temporally validated using the cohort of patients (*n* = 3325) discharged between 1st January 2016 and 30th June 2016 by the same hospital wards.

The AUC and the Net Reclassification Index (NRI) were calculated to compare the performance in risk prediction between the original and the simplified BRASS index and between the development and validation sets. The NRI represents the cumulative net proportion of the correctly reclassified events plus the net proportion of the correctly reclassified non-events.

The outcomes were available for all patients screened with the BRASS index and no imputation methods for missing data were applied.

The analyses were performed using STATA 13 and R.

## RESULTS

### Characteristics of the Training Set Population

The training set population counted 6044 patients. In this development sample, the mean age was 71.3 years and the males accounted for half (52%) of the sample. The most frequent reason for admission was the acute or chronic respiratory failure (5.8%), whereas the most frequent comorbidities were malignancy (18.2%), diabetes (14.7%), and hypertension (12.7%). Median LOS was 10 days (IQR, 6–16) and 196 patients were admitted in ICU during the hospitalization (Table S1, online supplement). In Table 1 are the listed patients’ characteristics according to the 10 items of the original BRASS index. About 72% of the patients were older than 65 years and half of them needed some kind of mobility support (mechanical/human assistance) or were not able to walk at all (51%). Four percent of the development sample lived alone with no social support and 4% of the patients came from nursing or residential care homes. Two thirds of the patients in the training set population had more than three active medical problems (67%), and 79% were taking more than three different drugs.

**Table 1:** Patient characteristics (original BRASS index items) of the training set population according to the discharge outcome.

	Discharged at home without complications	Complex discharge	Dead in hospital	Total
	(*N* = 3798)	(*N* = 1883)	(*N* = 363)	(*N* = 6044)
Age (years)				
≤ 55	689 (18%)	218 (12%)	18 (5%)	925 (15%)
56–64	531 (14%)	187 (10%)	29 (8%)	747 (12%)
65–79	1488 (39%)	766 (41%)	156 (43%)	2410 (40%)
≥ 80	1090 (29%)	712 (38%)	160 (44%)	1962 (32%)
Social support/living situation				
Lives only with spouse	1988 (52%)	797 (42%)	174 (48%)	2959 (49%)
Lives with family	860 (23%)	422 (22%)	72 (20%)	1354 (22%)
Lives alone with family support	658 (17%)	375 (20%)	57 (16%)	1090 (18%)
Lives alone with friends’ support	92 (2%)	84 (4%)	14 (4%)	190 (3%)
Lives alone with no support	107 (3%)	98 (5%)	7 (2%)	212 (4%)
Nursing home/residential care	93 (2%)	107 (6%)	39 (11%)	239 (4%)
Cognitive status				
Orientated	3261 (86%)	1252 (66%)	188 (52%)	4701 (78%)
Disorientated to some spheres some of the time	378 (10%)	360 (19%)	71 (20%)	809 (13%)
Disorientated to some spheres all of the time	48 (1%)	77 (4%)	8 (2%)	133 (2%)
Disorientated to all spheres some of the time	48 (1%)	68 (4%)	21 (6%)	137 (2%)
Disorientated to all spheres all of the time	49 (1%)	93 (5%)	32 (9%)	174 (3%)
Comatose	14 (0%)	33 (2%)	43 (12%)	90 (1%)
Mobility				
Ambulatory	2295 (60%)	614 (33%)	56 (15%)	2965 (49%)
Ambulatory with mechanical assistance	616 (16%)	305 (16%)	41 (11%)	962 (16%)
Ambulatory with human assistance	506 (13%)	395 (21%)	71 (20%)	972 (16%)
Non-ambulatory	381 (10%)	569 (30%)	195 (54%)	1145 (19%)
Sensory deficit				
None	2104 (55%)	933 (50%)	166 (46%)	3203 (53%)
Visual or hearing deficits	1413 (37%)	737 (39%)	136 (37%)	2286 (38%)
Visual and hearing deficits	281 (7%)	213 (11%)	61 (17%)	555 (9%)
Number of previous admission				
None in the last 3 months	2400 (63%)	994 (53%)	154 (42%)	3548 (59%)
One in the last 3 months	1053 (28%)	625 (33%)	144 (40%)	1822 (30%)
Two in the last 3 months	225 (6%)	181 (10%)	46 (13%)	452 (7%)
More than two in the last 3 months	120 (3%)	83 (4%)	19 (5%)	222 (4%)
Number of active medical problems				
≤ 3 Medical problems	1462 (38%)	474 (25%)	51 (14%)	1987 (33%)
Three to five medical problems	1382 (36%)	781 (41%)	149 (41%)	2312 (38%)
More than five medical problems	954 (25%)	628 (33%)	163 (45%)	1745 (29%)
Number of drugs				
Fewer than three drugs	929 (24%)	271 (14%)	30 (8%)	1230 (20%)
Three to five drugs	1301 (34%)	661 (35%)	120 (33%)	2082 (34%)
More than five drugs	1568 (41%)	951 (51%)	213 (59%)	2732 (45%)
Functional status				
In eating/feeding	541 (14%)	649 (34%)	194 (53%)	1384 (23%)
Bathing/grooming	913 (24%)	997 (53%)	245 (67%)	2155 (36%)
Toileting	951 (25%)	987 (52%)	248 (68%)	2186 (36%)
Transferring	1005 (26%)	1040 (55%)	254 (70%)	2299 (38%)
Incontinent of bowel function	429 (11%)	564 (30%)	170 (47%)	1163 (19%)
Incontinent of bladder function	551 (15%)	677 (36%)	191 (53%)	1419 (23%)
Meal preparation	962 (25%)	1000 (53%)	250 (69%)	2212 (37%)
Responsible for own medication administration	783 (21%)	841 (45%)	212 (58%)	1836 (30%)
Handling own finances	688 (18%)	759 (40%)	201 (55%)	1648 (27%)
Grocery shopping	902 (24%)	941 (50%)	233 (64%)	2076 (34%)
Transportation	1060 (28%)	1047 (56%)	249 (69%)	2356 (39%)
Behavior pattern				
Wandering	48 (1%)	66 (4%)	10 (3%)	124 (2%)
Agitated	75 (2%)	129 (7%)	36 (10%)	240 (4%)
Confused	219 (6%)	313 (17%)	78 (21%)	610 (10%)
Other	86 (2%)	150 (8%)	72 (20%)	308 (5%)

In Table 2 are the summarized three outcome categories and the discharge destinations of the patients. From the total training set population, 3798 (63%) patients were discharged at home without complications, 1883 (31%) had a complex discharge and 363 (6%) died during the hospital stay. A proportion of 8% of the total development sample was included in the complex discharge category because of a longer LOS (greater than the 90th percentile of DRG-specific LOS in the region), even if these patients were discharged at home.

**Table 2:** Outcome classification and discharge destination of the patients.

Discharged at home without complications	3798 (63%)
Dead in hospital	363 (6%)
Complex discharge	1883 (31%)
• Discharged at home with a longer LOS*	469 (8%)
• Skilled nursing facility (SNF)/residential care	713 (12%)
• Home care	88 (1%)
• Discharge against medical advice (AMA)	57 (1%)
• Transferred to another hospital	85 (1%)
• Transferred to another unit/facility	43 (1%)
• Inpatient rehabilitation facility (IRF)	342 (6%)
• Integrated Home Care Services (IHCS)	86 (1%)
Total	6044

*Patients discharged at home with a length of stay (LOS) greater than the 90th percentile of DRG-specific LOS observed in the Piedmont region hospitals. Median DRG-specific 90th percentile LOS was 20 days (IQR 11–30 days, max 157 days for heart transplantation)

### Predictive Ability of the Original BRASS Index

**Figure 1:**
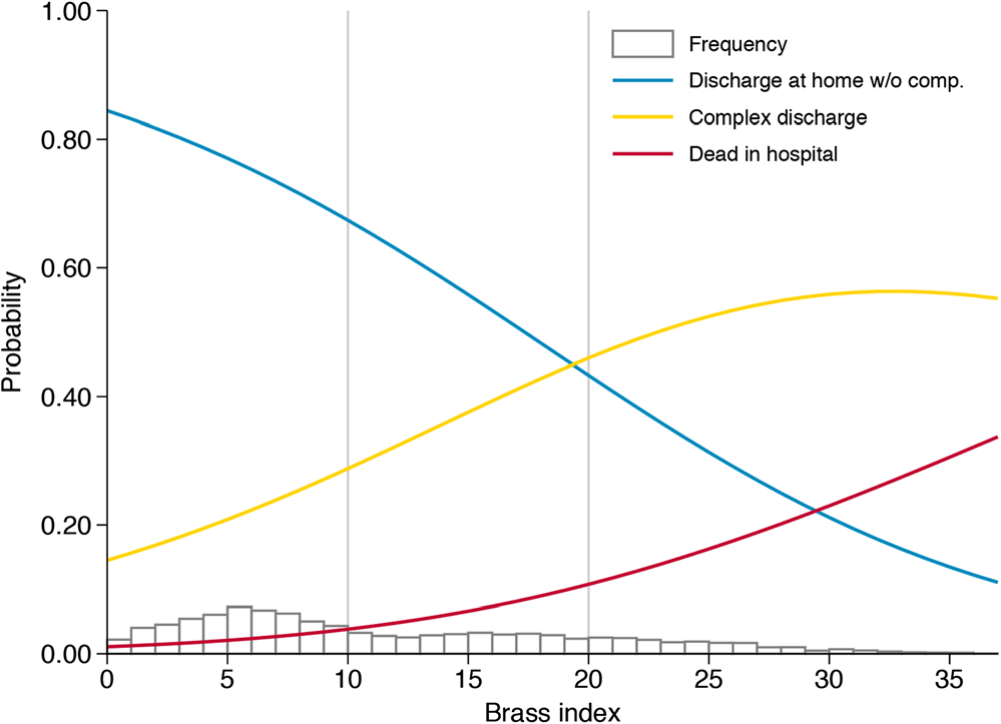
Estimated probability (multinomial logit model) of each discharge outcome and BRASS index.

The mean score of the original BRASS index was 11.37 (standard deviation [SD] = 8.07) for the whole cohort. Mean (SD) levels of the original BRASS index were 9.10 (7.00) for patients discharged at home without complications, 14.56 (8.11) for patients with a complex discharge, and 18.59 (8.4) for patients that died in the hospital. We found that the probability of complex discharge and hospital mortality increased proportionally to the BRASS index, (Fig. 1). The discriminative and predictive ability of the original BRASS index represented with the AUC were 0.70 (95%CI, 0.68–0.71) for complex discharge and 0.80 (95%CI, 0.78–0.83) for the hospital mortality. The analysis performed using a restricted cubic spline transformation of the BRASS index did not show any evidence of a non-linearity of effect on both outcomes.

### Model Derivation and Development of the New Simplified BRASS Score

**Figure 2:**
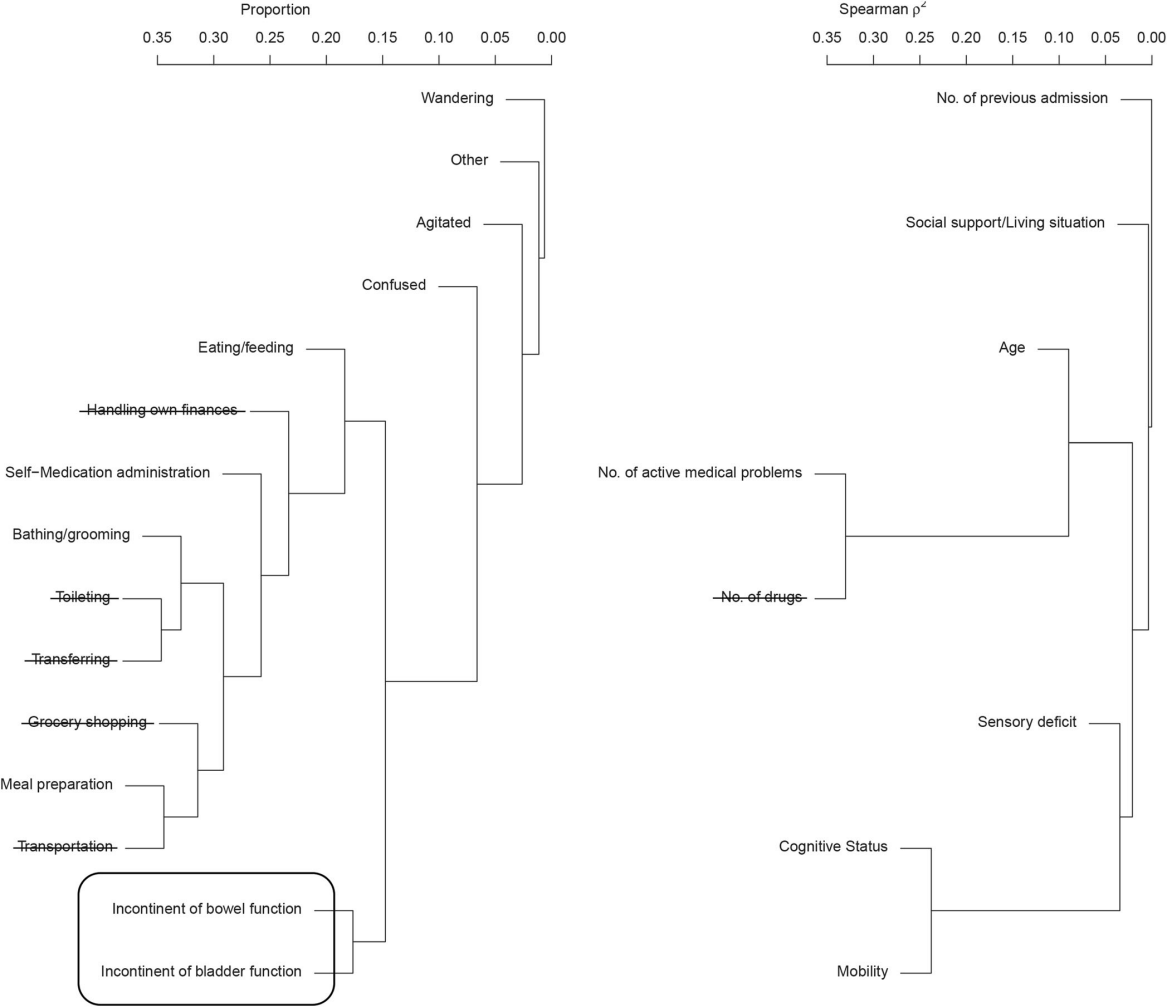
Data reduction process for dichotomous and ordinal variables using the proportion of both conditions present and Spearman ρ2.

Figure 2 is a graphical representation of the variable reduction process used to simplify the original index. The hierarchical clustering allowed the identification of strongly correlated variables and suggested which of the redundant items could be eliminated. For example, the two items “number of drugs” and “number of active medical problems” were strongly correlated, and it was decided to eliminate number of drugs from the model and to maintain only number of active medical problems for further analysis. Additionally, the item “number of drugs” was highly predictable by all other variables included (AUC = 0.810). Similarly, the following variables were eliminated: handling own finances (AUC = 0.977), toileting (AUC = 0.981), transferring (AUC = 0.979), grocery shopping (AUC = 0.972), and transportation (AUC = 0.977). The two items “bowel incontinence” and “bladder incontinence” were aggregated into a single variable “bowel and/or bladder incontinence.”

The final simplified model was presented with a new scoring, based on two different sets of weights, predicting either a complex discharge or hospital mortality (Table 3). The total sum points of the new, simplified BRASS index can range between 0 and 19 for the complex discharge outcome and from 0 to 28 for hospital mortality.

**Table 3:** Association of specific items with discharge outcomes. Original BRASS index weights (a), simplified BRASS model and modified weights (b).

	Original BRASS index	Complex discharge				Hospital mortality			
ITEM	Weight (a)	OR (95%CI)	*P*	Beta	Weight (b)	OR (95%CI)	*P*	Beta	Weight (b)
Age (years)									
≤ 55	0	1.00 (Ref.)			0	1.00 (Ref.)			0
56–64	1	0.91 (0.72–1.16)	0.443	− 0.094	0	1.52 (0.81–2.85)	0.188	0.421	2
65–79	2	1.16 (0.96–1.41)	0.128	0.149	0	2.25 (1.33–3.81)	0.002	0.811	3
≥ 80	3	1.01 (0.82–1.25)	0.92	0.011	0	1.98 (1.16–3.4)	0.013	0.685	3
Social support/living situation									
Lives only with spouse/family	0	1.00 (Ref.)			0	1.00 (Ref.)			0
Lives alone with family support	2	1.22 (1.05–1.43)	0.011	0.202*	1	0.91 (0.66–1.25)	0.559	− 0.096	0
Lives alone with friends’ support	3	2.35 (1.71–3.24)	< 0.01	0.856	4	2.07 (1.12–3.86)	0.021	0.730	3
Lives alone with no support	4	2.59 (1.93–3.48)	< 0.01	0.952	5	1.08 (0.49–2.41)	0.844	0.080	0
Nursing home/residential care	5	1.11 (0.81–1.53)	0.498	0.108	0	1.13 (0.72–1.78)	0.603	0.121	0
Cognitive status									
Orientated		1.00 (Ref.)			0	1.00 (Ref.)			0
Disorientated to some spheres	2	1.36 (1.15–1.62)	< 0.01	0.310	2	1.1 (0.80–1.52)	0.548	0.098	0
Disorientated to all spheres	4	1.42 (1.04–1.95)	0.027	0.354	2	1.71 (1.08–2.7)	0.022	0.535	2
Comatose	5	1.96 (1.02–3.76)	0.043	0.673	3	8.23 (4.22–16.0)	< 0.01	2.107	8
Mobility									
Ambulatory	0	1.00 (Ref.)			0	1.00 (Ref.)			0
Ambulatory with mechanical assistance	1	1.27 (1.06–1.54)	0.011	0.242	1	1.76 (1.13–2.75)	0.013	0.567	2
Ambulatory with human assistance	2	1.65 (1.35–2.02)	< 0.01	0.503	2	3.07 (1.99–4.74)	< 0.01	1.121	4
Non-ambulatory	3	2.61 (2.07–3.28)	< 0.01	0.959	5	7.16 (4.57–11.2)	< 0.01	1.968	7
Number of previous admission/ER visits									
None in the last 3 months	0	1.00 (Ref.)			0	1.00 (Ref.)			0
One in the last 3 months	1	1.24 (1.09–1.42)	0.001	0.217	1	1.69 (1.30–2.18)	< 0.01	0.522	1
Two or more in the last 3 months	3	1.39 (1.15–1.68)	0.001	0.330	2	1.96 (1.40–2.75)	< 0.01	0.674	2
Number of active medical problems									
≤ Three medical problems	0	1.00 (Ref.)			0	1.00 (Ref.)			0
> Three medical problems	1	1.14 (0.99–1.31)	0.077	0.128	0	1.52 (1.09–2.12)	0.014	0.419	2
Functional status dependence									
In eating/feeding	1	1.05 (0.88–1.26)	0.588	0.050	0	1.31 (0.96–1.81)	0.093	0.274*	1
Bathing/grooming	1	1.45 (1.17–1.79)	0.001	0.370	2	1.03 (0.67–1.57)	0.901	0.027	0
Meal preparation	1	1.23 (1.00–1.51)	0.053	0.204	1	1.29 (0.85–1.96)	0.238	0.253	0
Behavior pattern									
Agitated	1	1.54 (1.10–2.14)	0.011	0.429	2	1.59 (0.98–2.58)	0.062	0.462	2

The AUC representing the discriminative ability of the different outcome categories of the simplified and the original BRASS index were 0.71 (95%CI, 0.70–0.73) vs. 0.70 (95%CI, 0.68–0.71) for the complex discharge and 0.83 (95%CI, 0.81–0.85) vs. 0.80 (95%CI, 0.78–0.83) for hospital mortality, respectively.

The continuous NET reclassification index was 0.315 (95%CI, 0.262–0.368) for complex discharge compared to the discharge at home and 0.489 (95%CI, 0.419–0.559) for the hospital mortality compared to the discharged at home category.

### Temporal Validation of the Simplified Score

In the validation phase, a total of 3584 patients were recruited. After excluding 259 patients (7.2%) because of unsuccessful linkage with the hospital discharge records, a total of 3325 patients were included in the analysis. In this validation sample, the mean age was 71.19 years and the males consisted of 53% of the sample, similar to the development sample. The discharge destinations for the patients were also similar between the two samples, 2022 (61%) patients were discharged at home, 1120 (34%) had a complex discharge, and 183 (5%) died during the hospital stay.

The discriminative ability of the new simplified BRASS index did not differ meaningfully between the training and the validation sets. The AUC was 0.69 (95%CI, 0.67–0.71), not so different from the corresponding value in the training set (AUC = 0.71, 95%CI, 0.70–0.73) in discriminating patients at risk for discharge difficulties. A similar performance was observed also for the prediction of hospital mortality, with an AUC = 0.83 (95%CI, 0.81–0.85) in the training set and an AUC = 0.81 (95%CI, 0.78–0.84) in the validation set.

**Figure 3:**
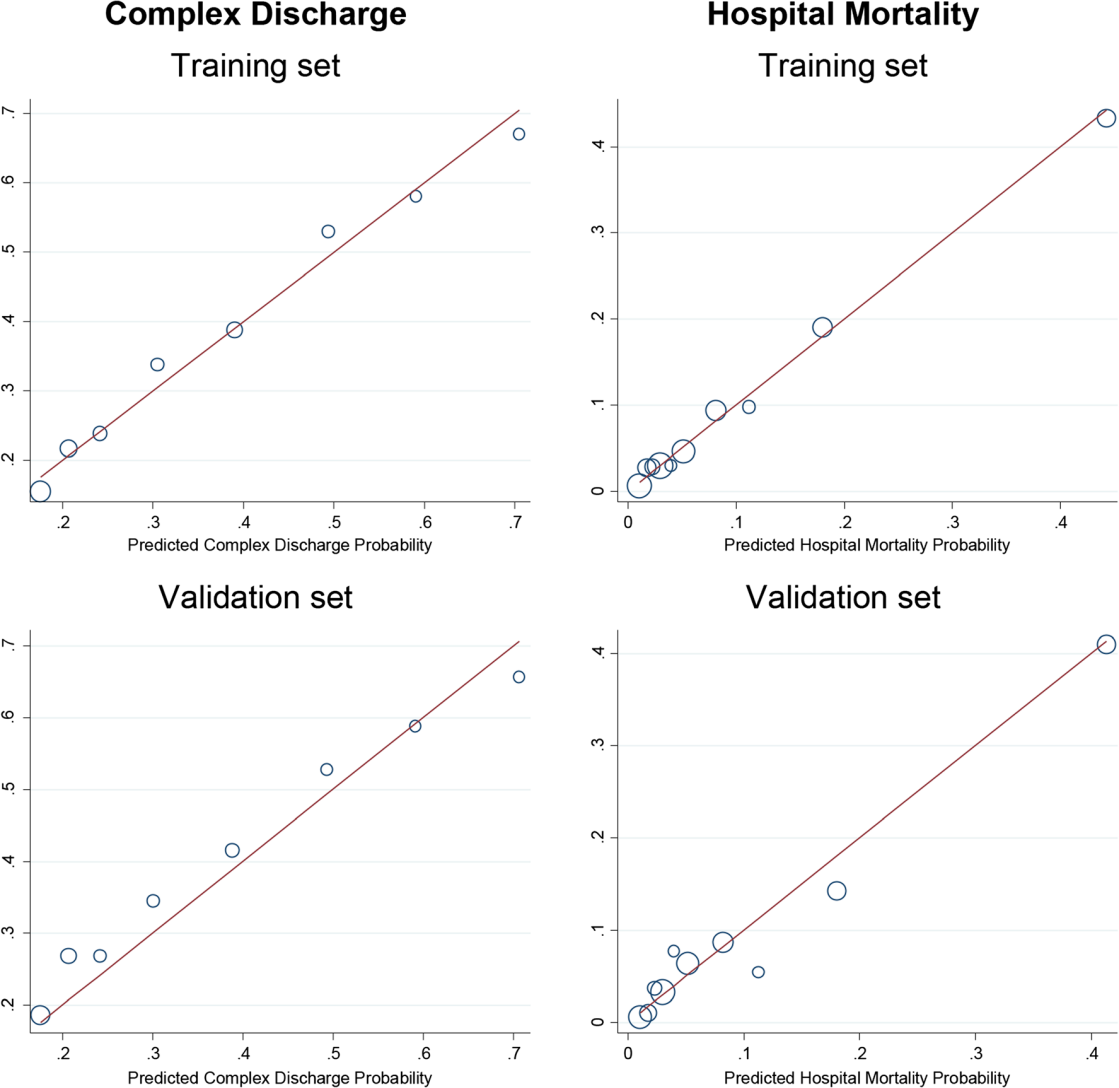
Calibration plot of predicted and observed outcome regarding the simplified BRASS index.

In Figure 3, the calibration of the simplified BRASS model was explored by plotting the observed to the expected frequency (predicted probabilities) of each of the two outcomes (complex discharge and hospital mortality) both in the training and in the validation set. The simplified BRASS model appears to be well-calibrated because each dot (group of predicted probabilities) fits close to the diagonal line of perfect calibration.

## Discussion

In this study, we developed and validated a new, simplified version of the original BRASS index, to improve the screening of patients at risk of complex discharge needing the activation of hospital discharge planning services. This tool showed also a very good prediction of hospital mortality. The results suggest that our simplified BRASS index has at least the same discriminative and predictive ability as the original BRASS index (AUC are 0.71 vs. 0.70 for the complex discharge and 0.83 vs. 0.80 for hospital mortality, respectively). Moreover, this simplified BRASS index has an additional advantage because it is based on a model with half the number of degrees of freedom (20 vs. 41) with respect to the original BRASS index, translated into a reduced number of questions (10 vs. 20), which means that it takes less time to be applied by the healthcare workers.

The predictive performance of the BRASS index was previously assessed in a number of studies. These experiences demonstrated that the BRASS index may be very useful in identifying patients who are at increased risk of prolonged hospitalization, in planning more appropriate discharges, and in reducing or preventing post-discharge problems.^9^^,^^11^^,^^12^

Mistianen et al. suggested the need of further research to better estimate the weights of the items of the BRASS index and to assess its predictive validity in a more homogenous population.^11^ Besides the reduction of the items, the main difference between the original and the simplified BRASS index is the re-weighting of the retained variables. For example, for predicting a complex discharge, the “non-ambulatory condition” increased its weight from 3 to 5 with respect to the original BRASS, whereas the “nursing home or residential care condition” completely lost its importance (from 5 to 0). Dal Molin et al. already raised some doubts about the item “Living in a nursing home/residential care” by reporting that in Italy, patients admitted from nursing homes/residential care facilities have a lower risk of prolonged hospitalization and complex discharge than other patients.^12^ On the contrary, the item “Age” of the BRASS index appears to be predictive only for hospital mortality, whereas it has no importance in the new context and appears to be no longer predictive for a complex discharge.

Dal Molin et al. mentioned that the BRASS index can be used for prediction of hospital mortality.^12^ However, to the best of our knowledge, our study is the first that analyzed complex discharge and hospital death as separate outcomes. We observed that the BRASS index had a strong, partially independent ability to predict hospital mortality that justified the estimation of a separate set of weights. Surprisingly, in our study, we found that the ability of the original and the simplified BRASS index in predicting hospital mortality is even better than the ability of predicting complex discharge (AUC = 0.83 for hospital mortality and AUC = 0.71 for complex discharge, respectively, for the simplified BRASS index).

In addition to the large sample analyzed, a remarkable strength of the present study is the temporal validation to test the performance of the simplified BRASS index. The temporal validation is a prospective re-evaluation of a new model, which is used to confirm its performance on a new sample from the same institution. It is considered external in time and thus intermediate between an internal and a true external validation.^13^ The performance of the new and simplified model in the new data was very close to the performance in the development set.

The availability of a validated screening tool to identify patients at risk for discharge problems may be helpful for improving the effectiveness of the discharge planning. We considered the simplified BRASS index to be a useful predictive instrument in clinical practice. However, in order for it to be routinely adopted as a predictive risk score, it must first be proven to be clinically effective to provide useful additional information to health care providers informing clinical decision-making based on patient outcomes.^13^ One of the future developments of this study is to identify an optimal cutoff point of the simplified BRASS index for the activation of the special hospital discharge planning service. A cluster randomized trial aimed at comparing two different cutoff points of the simplified BRASS index (corresponding to different workloads for hospital staff) in reducing the short-term readmissions to hospital, and the length of stay is now on-going in our hospital.

## Electronic supplementary material

Table S1 (DOCX 15 kb)
