# Supplementary material for: Development and validation of a simplified BRASS index to screen hospital patients needing personalized discharge planning
Source: J Gen Intern Med. 2018 Apr 16;33(7):1084–91. doi: 10.1007/s11606-018-4405-y (PMC6025690; doi:10.1007/s11606-018-4405-y)
Supplement: Supplementary file 1 — (DOCX 15 kb) [file 11606_2018_4405_MOESM1_ESM.docx]

**Table S1: Patient clinical characteristics**

| **Most frequent reasons for hospital admission** | N=6044 |
| --- | --- |
| Acute or chronic respiratory failure | 352 (5.8%) |
| Pneumonia | 315 (5.2%) |
| Heart failure | 287 (4.8%) |
| Stroke | 248 (4.1%) |
| Septicemia | 210 (3.5%) |
| Disorders of fluid electrolyte and acid-base balance | 204 (3.4%) |
| Chronic bronchitis | 152 (2.5%) |
| Hypertensive heart disease | 114 (1.9%) |
| Chronic liver disease/Cirrhosis | 111 (1.8%) |
| Hypertensive heart and chronic kidney disease | 92 (1.5%) |
| Acute pulmonary heart disease | 73 (1.2%) |
| Cardiac dysrhythmias | 73 (1.2%) |
| Cholelithiasis | 59 (1.0%) |
|  |  |
| **Comorbidities** |  |
| Malignancy | 1098 (18.2%) |
| Diabetes | 886 (14.7%) |
| Hypertension | 770 (12.7%) |
| Heart failure | 541 (9%) |
| Coronary artery disease | 418 (6.9%) |
| Chronic liver disease/Cirrhosis | 292 (4.8%) |
| Dialysis-dependent kidney failure | 16 (0.3%) |
|  |  |
| **Length of stay, median days (IQR*)** | 10 (6-16) |
|  |  |
| **ICU** during the index hospitalization** | 196 (3.2%) |

*IQR- Interquartile range

**ICU- Intensive care unit
